# The Thin Line Between Waking and Sleeping in Athletes: A Call for Yoga Nidra in the Sporting Context

**DOI:** 10.3389/fpsyg.2021.654222

**Published:** 2021-05-21

**Authors:** Selenia di Fronso, Maurizio Bertollo

**Affiliations:** Department of Medicine and Aging Sciences, Behavioral Imaging and Neural Dynamics Center, University “G. d'Annunzio” of Chieti-Pescara, Chieti, Italy

**Keywords:** arousal management, emotional balance, mind-body, recovery, relaxation, restoration, self-regulation, training

## Introduction

Yoga is an umbrella term that refers to a series of activities including different holistic exercises (e.g., balance, stretching, breathing exercises) and diverse styles of practice (Cramer et al., 2016). Regarding styles, yoga can vary from a more physical practice, such as ashtanga yoga, to a more meditation-based practice, such as Yoga Nidra (YN; literally yoga sleep). YN was developed in 1976 as a relatively easy-to-learn meditation practice to be used by various people regardless of their previous experience (Moszeik et al., 2020). Unlike other meditation-based practices (e.g., transcendental meditation, body scan), considered as aware awake states, YN is rather considered as an aware sleep state (Sharpe et al., 2021). More precisely, this practice, executed in supine position, naturally stimulates a hypnagogic state wherein an individual is physiologically asleep yet maintains an internal/external awareness (Sharpe et al., 2021); there is a withdraw from other senses, and only the auditory channel is open so that the participant stays aware of the directions coming from the instructor, but practices detachment from all other stimuli. YN interventions have been associated with significant improvements in sleep parameters such as sleep onset latency and sleep quality (Datta et al., 2017; Moszeik et al., 2020) because of a general parasympathetic dominance (Markil et al., 2012) and a subsequent high cardiac vagal control (Werner et al., 2015; see also *YN Effects and Potential Benefits on Athletes*); it first stimulates the parasympathetic nervous system increasing heart rate variability (HRV), or its high-frequency components (Markil et al., 2012), and alpha waves, to then demonstrate the symptoms of deep, non-REM sleep, including theta and delta brain waves (Parker et al., 2013).

One of the core components of YN sessions is represented by a personal resolution called Sankalpa, the Sanskrit word for “intention.” This resolution is expressed as a simple, short, and positive sentence (e.g., “I am calm,” “I am successful”), which is repeated in the beginning and in the end of the session. Its regular mental repetition “drives” the unconscious toward the desired state by stimulating cognitive restructuring processes (Moszeik et al., 2020). Encompassing a sequence of guided body awareness, visualization, and breathing exercises, YN is also described as a complete and systematic method of inducing physical and mental relaxation achieved by turning inward, away from most of outer experiences (Saraswati, 2009; Parker et al., 2013). For a more comprehensive description of YN stages, see Table 1.

**Table 1:** The different stages of Yoga Nidra.

1	Sankalpa: A resolution, an *intention* that the participant needs in his/her life
2	Rotation of consciousness: Awareness to the different body parts, without concentrating
3	Breath awareness: Attention to the breathing that should not be altered or forced in this stage
4	Feelings and emotions: Intense sensations are awakened and then “removed”
5	Visualization^*^: In this stage, the participant should enter the hypnagogic state
6	Ending: The sankalpa is repeated, and the participant slowly comes back to the reality

*Sankalpa: the Sanskrit word for “intention” expressed as a simple, short, and positive sentence (e.g., “I am calm,” “I am successful”)*.

* *This part can be guided in detail (describing, for example, peaceful places) or be more open, encouraging the participant to visualize personal functional places or moments. Stages 2, 3, and 4 are functional to relaxation and facilitate the hypnagogic state; sounds can be used to make the auditory channel remain active*.

### YN Effects and Potential Benefits on Athletes

YN showed positive effects on several variables and conditions. For example, such practice was found to benefit addictive behaviors, pain, and conditions related to cardiovascular diseases (e.g., Stankovic, 2011). YN, by means of Sankalpa and cognitive restructuring processes, may prevent and/or counteract dysfunctional cognitions; this in turn stimulates life satisfaction and personal well-being (Diener et al., 1999). Importantly, the practice induces a hypothalamic response, stimulating parasympathetic and suppressing sympathetic nervous system activity (Dol, 2019). Accordingly, YN down-regulates hyperarousal and stress-related biological indices, such as skin conductance and cortisol levels (Kumar and Joshi, 2009). As a long-term effect, the shift toward parasympathetic dominance and the subsequent high cardiac vagal control may increase slow-wave sleep and improve both subjective and objective sleep quality (Werner et al., 2015). The more the parasympathetic activity dominates during sleep, the more the sleep is restorative and rejuvenating, and body recovers balancing the daily stress responses (Datta et al., 2017). Thus, in more recent years, there has been a growing interest in YN effects on depression, perceived stress, posttraumatic stress disorder, well-being or quality of life, and insomnia/chronic sleep disorder, especially considering students, workers, and veterans; for a more detailed overview on these topics, see https://www.irest.us/research.

Notwithstanding the increasing number of studies on the influence of YN on stress or sleep, there is still scant research concerning this practice in the sporting context. From an applied standpoint, yoga stretching, biofeedback and pure meditation, breathing, and imagery are the most frequently mentioned techniques used by athletes to foster inner balance and recovery (Pelka et al., 2016). Athletes yet face unique physiological and psychological stressors daily, which may contribute to injuries, overtraining, burnout, and/or other physical and mental health issues. Accordingly, they would need continuous exploration of more complete strategies to counteract physical and mental tension and other detrimental stressor effects. Moreover, sleep quality, proven to be increased by YN (e.g., Moszeik et al., 2020), is one of the essential parameters to improve recovery–stress balance in athletes (Loch et al., 2019). Only recently, this technique was used in the archery context and was found to aid in better athletic performance not only directly by enhancing vigilance but also indirectly by increasing training participation due to better physical and mental recovery (Datta et al., 2020). Drawing on these notions and on the principles of translational medicine aimed at improving health outcomes, both research and applied field should rely more on this mind–body practice as a key strategy for a complete and adequate recovery. In the sporting context, adequate recovery includes not only social but also psychological and physiological activities, which can be either active or passive behaviors (Loch et al., 2019). Of note, most of the athletes may prefer movement and active rather than static practices (Goodman et al., 2014). However, YN is not a solely supine calming posture. Indeed, 1 h of practice is judged as restorative and rejuvenating as 4 h of ordinary sleep (Saraswati, 2009). This feature would be of utmost importance in the sporting field. Indeed, while athletes may require more sleep compared to non-athletes to recover from training and competition demands, sleep deprivation is quite common among them. YN might improve sleep quality, as mentioned previously, and help athletes to deal with sleep deprivation. For example, because of its effects on sleep latency (Datta et al., 2017; Moszeik et al., 2020), exploring this practice might help athletes with the “sleepability” (the ability to nap on demand) that could be used as a strategic skill to manage sleep challenges and debt (Gupta et al., 2020). These could be due to, for example, travel fatigue and jet lag as athletes are often required to embark on long travel for competition reasons (e.g., van Rensburg et al., 2020). Better sleep would also benefit arousal management; this may reduce stress reactivity, which is caused by the arousal that leaves one overly reactive to stimuli (Park et al., 2020). This reduction may be crucial for health purposes, especially in athletes as they continuously face training and competition stimuli. Better arousal management might also be indicated for not losing appropriate activation levels essential to sport performance (Pelka et al., 2016). Additionally, improved sleep and arousal management along with reduced pain scores and muscle tension, other outcomes of YN, would constitute a virtuous cycle (Sutar et al., 2016), which could lower the possible “threats” of training and competitions on the nervous system. Further, a YN session could replace a traditional nap, stimulating even more vivid and clearer experiences than those that occur in the traditional nap modality, and in light of reduced muscle tension, it can lead to lucid dreaming (see, for e.g., Cebolla and Cheron, 2019).

There is mounting evidence that self-regulation skills are among the most important aspects underlying athletes' performance and well-being (di Fronso et al., 2020). Of note, the regulation of feelings, thoughts, and actions plays an important role not only before and during but also after performance. Accordingly, recovery can be considered as a self-regulation process too (Kellmann et al., 2018). Recovery self-regulation is conceptualized as the identification of athletes' current and desired future state and the implementation of actions to minimize the discrepancy between both states during recovery (e.g., between competitions or training sessions; Balk and Englert, 2020). In this scenario, YN practice may stimulate essential processes, such as self-monitoring of feelings and thoughts to be regulated, and the achievement or replenishment of desired physical, cognitive, and emotional resources. Such processes would be driven by the perfect combination of awareness and resolution exercises undertaken during absolute relaxation conditions (Saraswati, 2009). Moreover, the physical and mental detachment from sport-related requests experienced throughout the practice may represent the “switch-off” that athletes need to attenuate the adverse effects of high demands on health and well-being (e.g., Sonnentag and Fritz, 2015). Also, cognitive restructuring processes induced by YN practice may regulate postperformance negative emotions, thus stimulating emotional detachment (Balk and Englert, 2020). It is indeed essential that athletes distance themselves from post-performance emotions (e.g., anger) that can hinder recovery and that can increase arousal, thereby causing energy depletion (e.g., Loch et al., 2019). Positive emotions would instead stimulate the production of hormones, such as dopamine, capable of lowering the stress response and promoting adequate recovery (Balk and Englert, 2020).

## Discussion

YN practice has peculiar and established physical as well as mental benefits (e.g., Moszeik et al., 2020), which might also lead to a beneficial impact on athletes' recovery (and well-being) in terms of sleep quality, arousal management, and self-regulation processes. However, considering both research and applied field, YN should still be promoted among athletes. In this regard, future research and applied directions for sports professionals could be of prominent interest.

From an applied point of view, athletes might be suggested to adopt YN as a constant training or competition recovery strategy (i.e., to execute—alone or after other exercises—between training sessions or competitions, e.g., Pelka et al., 2016). This practice may serve during rest periods not only as a physical but also as a mental recovery strategy to counteract the effects of multiple training bouts on a single day or tight competition bouts over weeks and restore the system and mental balance (Loch et al., 2019). Generally, a YN session would last about 1 h; however, given time is a scarce resource, especially in sports venues and between training sessions, the practice can be temporally adapted with sessions lasting 15 min. This temporal adaptation would still guarantee a systematic physical and mental relaxation and restoration (Saraswati, 2009). Importantly, as athletes may fall asleep instead of maintaining “the thin line between waking and sleeping,” the first experiences of YN should not exceed 45 min. After this time, the brain enters the sleep modality, and it would be necessary to complete four to five cycles of normal sleep to properly recover. On the other hand, advanced YN practitioners, after 45 min, can access theta and delta wave states with full consciousness (Parker et al., 2013), ultimately aiming for a super-conscious sleep experience. A constant practice and an instructor with theoretical and practical knowledge would be the keys to not fall asleep and to sleep quality and emotional balance improvement. Thus, not only athletes but also practitioners may try to broaden their horizons and learn new mind–body techniques strengthening their holistic approach to sports coaching.

Moreover, sports professionals can encourage athletes to take a YN “nap” instead of traditional ones (using, for example, taped instructions) while taking advantage of travel time (e.g., van Rensburg et al., 2020). Of note, as recovery is a highly individual process, this strategy should be applied according to individual sleep needs and requirements (Loch et al., 2019). Given that YN would also strengthen learning processes (Sharpe et al., 2021), crucial especially in young athletes, it might be suggested to briefly review tactics or other key training factors and match-related information immediately before YN. Likewise, in accordance with a more open practice characterized by less detailed visualization instructions, the same information can be visualized into stage 5 of the practice (Table 1).

From a (qualitative/quantitative) research point of view, randomized controlled trials adequately powered, comparing athletes of different sports (e.g., precision vs. endurance), as well as different mind–body techniques to foster recovery, would be useful. Participants would complete surveys and questionnaires (that would be helpful in evaluating subjective benefits of such practices) and would undergo a psychophysiological assessment [e.g., electroencephalogram (EEG), electrocardiogram (ECG) data collection; di Fronso et al., 2017, 2020]. From a neurophysiological perspective, as individual alpha peak frequency shifts can occur because of physical effort (e.g., Gutmann et al., 2018), scholars should consider establishing the impact of YN after training or competitions on the alpha peak modulation (di Fronso et al., 2019). This would allow to better examine neural correlates of a passive recovery strategy. From a cardiophysiological perspective, as most of the studies reveal that relaxation produces a transient effect on HRV and long-term relaxation studies are scarce, ECG data and *ad hoc* trials could be used to clarify the nature of changes in HRV, as well as the physiological mechanisms underlying the increase in HRV induced by YN (Markil et al., 2012). Findings, while providing clearer data concerning YN mechanisms, may further shed light on the effect of an aware sleep state on sleep quality and on the usefulness of this kind of yoga technique for improving recovery in athletes.

## Author Contributions

All authors listed have made a substantial, direct and intellectual contribution to the work, and approved it for publication.

## Conflict of Interest

The authors declare that the research was conducted in the absence of any commercial or financial relationships that could be construed as a potential conflict of interest.
